# Communication, socialization, and ITC. The psychosocial construction of sustainability

**DOI:** 10.3389/fpsyg.2023.1277577

**Published:** 2024-01-05

**Authors:** Enric Pol, Angela Castrechini-Trotta, Isabel Pellicer-Cardona, Cristina Cañete-Massé

**Affiliations:** ^1^Department of Social Psychology and Quantitative Psychology, Faculty of Psychology, University of Barcelona, Barcelona, Spain; ^2^Social, Environmental and Organizational Psychology Research Group (PsicoSAO), SGR 290 Catalonia Government, Barcelona, Spain; ^3^Research Group on Interaction and Social Change (GRICS), SGR 233 Catalonia Government, Barcelona, Spain; ^4^Psychology, Sciences of Education and Sport, Blanquerna, Ramon Llull University, Barcelona, Spain

**Keywords:** sustainability, environmental communication, environmental education, socialization, mass media, social media, ITC

## Abstract

Over the past two decades, the facets related to environmental crises (in the plural) have grown increasingly intricate. What began as environmentalists’ apprehension over nature degradation and the encouragement of citizen-driven initiatives has evolved. The current shift in emphasis and prevailing message strives to foster a culture where citizens refrain from independent initiatives. Instead, the directive is to heed the guidance of the knowledgeable (scientists, politicians, corporations, interest groups, etc.), as substantiated by our investigative findings, which align, in part, with existing literature. Conversely, our exploration into environmental communication, notably the insights gleaned from longitudinal research concerning pro-environmental knowledge, attitudes, and actions, reveals a decline in citizens’ effective inclinations toward embracing pro-environmental behaviors. Meanwhile, the escalation of the climate crisis is fueling heightened levels of echo-anxiety and solastalgia. This trajectory is closely intertwined with a growing global disillusionment within society – *not just regarding the future* – instilling a sense of disillusionment concerning pro-environmental messages and slogans disseminated by governing bodies and corporations. This has led to a state resembling learned helplessness, as articulated by Seligman, or what we prefer to term “induced” helplessness, rather than fostering conditions conducive to empowerment. This article comprehensively examines various reports and our inquiries, revealing how communication management and its constituents lie at the heart of forging novel narratives, fresh cognitive dissonances, and emerging social representations. Notably emphasized is the pivotal role played by information and communication technologies (ICT), particularly through dissemination on widely-used social networks. Since the 2010s, these platforms have assumed a paramount role in shaping socialization processes, surpassing educational institutions and conventional mass media.

## 1 Introduction

Undoubtedly, the information disseminated by *mass media* wields a defining influence in the societal shaping of reality, as posited by Berger and Luckman (1966), referring to a collective imagination that serves as a crucial touchstone for individuals and social collectives, steering their choices. These news items are scrutinized as narratives, discourses, imaginaries, and representations. While various theoretical and epistemological frameworks employ different terms to address a similar “subject” of study, they invariably entail distinct conceptualizations to elucidate their functions. An illustrative instance lies in the assorted perspectives on social influence explored by seminal figures in social psychology, such as Ash, Sheriff, and Milgram, among others of note. Further contributing to this realm is Moscovici’s theory of Social Representations (SR), which holds considerable sway in the Francophone and Latin American environmental psychology domains, though its presence is comparatively less pronounced in the Anglophone sphere (yet still extant). This compels us to confront the initial inquiry we intend to address with our data: What portrayal of Environment, Sustainability, Climate Change, and similar topics have the mass media constructed? Additionally, who are the primary touchpoints facilitating the comprehension of pro-environmental conduct – or the inclination toward specific behaviors – among individuals and society?

The second research query directs our attention to the psychological mechanisms within these processes. One such exemplar is the cluster of theories stemming from Festinger’s experimental groundwork on “cognitive dissonance” (Festinger, 1957). Festinger’s insights guide us to the deduction that human beings tend to be more “rationalizing” than strictly “rational” (Fointiat, 1998; Fointiat et al., 2013). Furthermore, this perspective has engendered a line of investigation centered on creating scenarios that elicit cognitive dissonance. This approach aims to induce contradiction within individuals, compelling them to reevaluate their stance and conduct through the discomfort experienced, ultimately fostering a shift in perspective and behavior (Fointiat et al., 2011).

An additional dimension pertains to the contributions that cognitive and developmental theories proposed by figures such as Piaget, Vygotsky, Bruner, and Erikson, as well as their respective followers, can furnish. Furthermore, the theories of social influence (as previously mentioned) also play a pertinent role. Another realm of relevance encompasses the theories concerning attitudes and their alteration, as proposed by Thurstone (1931), Allport (1935), and Heider (1946, 1958), among others. Those theories that endeavor to effect behavior modification are of particular significance, with a pronounced emphasis on environmentally conscious or sustainable behaviors (Steg and Vlek, 2009, among others). Additionally, it’s imperative to consider viewpoints that address shifts in environmental behavior, often associating it predominantly with formal education while occasionally overlooking the pivotal role of socialization – both from a psychosocial and psychoeducational standpoint.

This article delves into select findings derived from our original research encompassing diverse facets linked to these intricate processes. Our scrutiny extends to assessing the degree to which present dynamics bolster empowerment or, conversely, foster circumstances of “induced” learned helplessness (in alignment with Seligman’s formulation). Furthermore, we present the latest insights from a longitudinal study on knowledge and inclinations toward sustainable behaviors. This study was conducted across three time points: 2006–2007, 2014, and 2022–2023. We examine the repercussions of these findings on the core concepts previously elucidated. Rooted in these data, we will explore how the role of information and communication technologies (ICTs) and social networks rejuvenates certain concepts that have experienced waning prominence in psychology over recent decades, notably social influence processes and socialization mechanisms.

## 2 The environment in the mass media

Throughout the initial two decades of the 21st century, sustainability emerged as a novel and affirmative social value, permeating various advertising campaigns. These encompassed commercial ventures, institutional initiatives, and those with an overt pro-environmental focus originating from various sources. The climate emergency, although infrequently addressed – primarily coinciding with the convening of COPs^1^ – was more often framed in the relatively moderate term ‘climate change’ than the more urgent term “climate emergency.” From the Earth Summit of Rio ‘92, where emphasis was placed on three high-risk aspects: desertification, loss of biodiversity, and climate change, the last one has gained increasing relevance as an alert and emergent element. The first COP in Berlin in 1995 solidified climate change as a priority problem. This momentum led to COP-3 in Kyoto in 1997, formulating the first major protocol for controlling emissions. Subsequent milestones include COP 21 in Paris (2015) and COP 26 in Glasgow (2021), which committed to reducing emissions by 30% by 2030 and halting deforestation. Despite the growing urgency to address climate change, the COP27 held in 2022 in Sharm el-Sheikh and the COP28 held in 2023 in Dubai were marked by inconsistencies and contradictions regarding objectives, format, settings, and the utilization of natural resources during the conferences.

The COPs evolution has brought both relevance and visibility to climate change as an element of alert and emergency. However, the credibility of the messages and slogans has been undermined by the uncertain outcomes and performance of the agreements among participating countries. Simultaneously, these meetings have been accompanied by demanding social movements, occurring in parallel gatherings and occasionally even participating within the COP (refer to Postigo et al., 2013). Among these movements, perhaps the most emblematic case was Greta Thunberg’s presence at COP 24 in Katowice, Poland, in December 2018, along with some subsequent interventions. These movements play a pivotal role in introducing fresh perspectives and new narratives, such as the concept of “climate justice.”

The burgeoning global movement for climate justice possesses a robust social foundation capable of coordinated mobilization in what are termed “counter-summits,” as well as decentralized actions across various regions worldwide (Borràs, 2016). A prime instance is the Fridays for Future movement, a global climate initiative advocating systemic transformation and urging politicians to fulfill their responsibilities. Regrettably, these actions have sometimes been employed in the media, or at least by certain outlets, to undermine these movements and individuals. According to Borràs (2016), the prevalence of adult perspectives in media coverage can disempower young protestors by deconstructing the political nature of their agenda and demands. A study conducted in Germany (Von Zabern and Tulloch, 2021) unveiled that media portrayal tends to fortify existing power dynamics, depicting young protestors as being manipulated by adult interests. This exclusionary stance can impede the political efficacy of young individuals, further contributing to their sense of disempowerment.

Contemporary media faces the formidable task of aptly communicating the escalating information and intricacies inherent in environmental concerns. The deficiency in awareness and comprehension regarding the challenges and potential remedies poses a hurdle for individuals, organized collectives, and governments to undertake substantial and resolute actions to achieve efficacious socio-ecological adaptation (CEIA-Centre d’Estudis d’Informació Ambiental, 1999).

The media wields a significant influence in shaping this paradox. Nonetheless, despite the surge in extreme weather events like droughts, hurricanes, floods, or fires, many individuals do not always perceive an immediate impact. Multilateral conferences might be remote and detached, while scientific models projecting sea-level rise or migration can prove challenging to grasp. Furthermore, the media grapples with the added complexity of making climate change coverage engaging for diverse segments, encompassing youth, “believers,” and those currently disinterested in the subject (Newman et al., 2021). However, we must not overlook the phenomenon Uzzell (2004) termed environmental hypermetropia: an apparent profound concern for global issues coupled with an inability to recognize and shoulder the responsibility for nearby environmental challenges, which necessitate personal involvement and action.

However, this scenario has also evolved over time, bringing into prominence three relatively new terms: eco-anxiety, solastalgia and ecofatigue. The first concept encompasses the emotional responses individuals experience when confronted with environmental issues, whether directly or indirectly, including through exposure to news and media coverage (Albrecht, 2011). The second concept, solastalgia describes the distress that is produced by environmental change impacting on people (Albrecht et al., 2007). The third concept, eco-fatigue, also known as green fatigue, arises from an overwhelming sense of responsibility or guilt stemming from excessive environmental information or pressure. It serves as a coping mechanism to avoid perpetual anxiety, leading to a tendency to detach oneself from environmental issues (Pol, 2000; Pol and Marchand, 2023). These concepts represent the various ways in which individuals grapple with the environmental challenges of our time, highlighting the multifaceted impact of environmental concerns on human well-being.

Although traditional newspapers and mass media serve as the primary conduits of environmental information, they are often regarded as offering subpar information quality. Despite this, numerous studies and surveys have indicated that they retain the highest level of credibility as an information source (CEIA-Centre d’Estudis d’Informació Ambiental, 1999; Valencia et al., 2010; Newman et al., 2021). It becomes necessary to ponder what constitutes a source’s “actual” and perceived quality or trustworthiness. It is crucial to acknowledge that according to Festinger, one of the fundamental inclinations among individuals is to evade cognitive dissonance. A classic response to this is disregarding or negating information that contradicts personal beliefs unless compelled otherwise.

Consequently, individuals tend to gravitate towards media outlets that echo their existing viewpoints, reinforcing their chosen stance. In essence, if the information is presented by “my” newspaper, “my” radio, “my” television, or within “my” social networks, it is deemed truthful (instilling trust). Conversely, if a source is not perceived as “mine” or aligned with one’s affiliations, the information is dismissed as falsehood and labeled fake news. In simpler terms, the medium that resonates with one’s preconceived notions is the one they place faith in.

This prompts us to delve into a comprehensive examination of environmental communication. We approach this task from the vantage point of communication sciences and the psycho-socio-environmental standpoint. We draw upon findings from two of our research endeavors to illuminate this endeavor. These investigations aim to scrutinize the treatment of environmental matters within specific media outlets. Before delving into these research outcomes, it is essential to outline the underlying processes from the purview of communication sciences.

## 3 Mass media, social media, and environmental education: influences and effects

The study of environmental communication and the exploration of the environmental impacts of various technologies intertwined with the progression of conventional mass media and social networks has garnered substantial interest across diverse realms of knowledge (Bergillos, 2020, 2021). Furthermore, the landscape of communication processes has experienced profound transformations after the global COVID-19 pandemic. These changes encompass not only the utilization of new technologies by citizens but also extend to the aesthetics and manner in which messages are conveyed. This pertains to the desired content and the orchestrated presentation to convey these messages (Deuze, 2020).

As demonstrated by Bergillos (2021), the presence of media is ubiquitous, exerting influence over both social environments and individuals. Simultaneously, these media are shaped by societal influences. Despite this intricate interplay, many aspects regarding their roles, influences, and consequences remain relatively unexplored. Furthermore, the emergence of virtual networks and novel technologies integrated into traditional media has significantly transformed their functions and impacts. This shift affects their operational mechanisms and the resulting content, thereby shaping the construction of new realities.

For instance, as highlighted by the author, the issue of climate change has fostered a pronounced polarization within the information ecosystem. This has led to an increase in misinformation, contradictions, and ambivalence surrounding the origins and repercussions of climate change, as well as the roles of individuals and mass media in shaping human behavior and societal dynamics. Opel (2015) elaborates on this point, elucidating that from a communication sciences perspective, *mass media* not only provide information about the environment but also contribute to establishing its value and significance. This aligns with the longstanding observations of social psychology spanning over a century.

Similarly, Bergillos (2021, p3) suggests that we must reflect on the leading role of communication sciences:

“*In an uncertain context that is being shaped by invisible phenomena, such as climate change and media effects … we have to face the contradiction of the supposed human influence that has transformed the Earth’s ecosystems and the powerlessness of not knowing to what extent our activities are involved in this transformation, matching the ambivalence of the presumed media presence and influence in people’s lives*.” (Bergillos, 2021, p. 3).

The role of agency in environmental matters, encompassing both human and non-human elements, has gained increasing attention in communication sciences, reflecting the consequences of diverse communication management strategies. This engrossment extends to the realms of social and behavioral sciences. Indeed, the prevalence of environmental-related information in media has surged, concurrently accompanied by persistent depictions of nature on our digital screens, as underscored by Opel (2015). While media serves as a conduit of environmental awareness, it concurrently assumes the role of attributing significance and interpretation to it. This dual role is articulated by Ruiz (2015), who characterizes communicators as “symbolic architects.”

Castrechini (2008) accentuate the necessity for research into the interplay between “representations” and reality, encompassing the environment and the impact of its portrayal as news. This inquiry extends to probing the environment’s role within popular culture, questioning whether this dynamic represents genuine interconnectedness or constitutes a form of “environmental fiction.” Furthermore, communication is a pivotal component of facilitating public engagement in environmental decision-making processes, and it holds the potential to address and dissect the complexities of climate change, as underscored by Boykoff et al. (2015, 2020) and Pezzullo and Cox (2018).

Communication systems have undergone significant changes, and in conjunction with these evolving networks, the monitoring of messages and the construction of meanings have grown increasingly intricate. In addition to this, as noted by Silverston (2010), it is imperative to acknowledge that within the digital media realm, encountering the “Other” proves to be an elusive endeavor. The “Other” appears on my screen, blurring the lines between absence and presence, making the distance feel near. As elucidated by Silverston (2010, p. 27), this spatial disconnect erodes the anticipated sense of responsibility among individuals.

Gladwell (2011) accentuates how the emergence of novel communication technologies, particularly within social networks, has fostered an environment where concise and uncomplicated messages prevail. This environment tends to favor the establishment of “weak ties,” as underscored by Gladwell (2011), which, regrettably, fails to cultivate robust activism or strategic deliberation.

Aran-Ramspott et al. (2018, 2022) have delved into media and networks’ role in socialization. In tandem with Jenkins et al. (2016), they expound on how the youth are presented with an increasingly expansive array of choices, ranging from devices and screens to social networks and applications, all of which they access at ever-younger ages. Furthermore, spanning various formats over time and technological advancements, the identification of preadolescents as a distinct market segment dates back to the 1980s (Ekström and Tufte, 2007). Given their heightened susceptibility to environmental influences in shaping their sense of self (Bernete, 2010), adolescents necessitate a comprehensive grasp of their interactions within the digital landscape (Blomfield Neira and Barber, 2014). Nonetheless, a research gap remains concerning the role of “influencers” as potential guides in the intricate socialization processes and identity formation among preadolescents.

Beyond the socialization role assumed by social networks, it is equally imperative to consider the significance of Environmental Education (EE) within the educational milieu, spanning schools and universities. EE is pivotal in enhancing environmental knowledge and fostering positive attitudes towards ecological concerns (Duerden and Witt, 2010; van de Wetering et al., 2022).

As per Díaz-Pont and Tarragona’s assessment (2003), educational programs addressing the environment and sustainability stand as the primary wellspring of environmental knowledge. Following this, social networks and other media contribute, as Mühlhäusler and Peace (2006) noted. However, as we have just seen, other authors point to the dominant role of networks, and certain authors emphasize the imperative to rejuvenate EE programs to foster more profound, experiential and standardized learning engagement.

The pioneering environmental education of the 1970s, once considered a “revolutionary” undertaking of nascent environmental movements, has since been institutionalized in the late 1980s, 1990s, and beyond. This transition has undoubtedly brought about changes in both the content and strategies employed, as well as the sources and promoters of environmental messaging. These changes have raised concerns about the credibility and trustworthiness of broadcasters and their messages (Newman et al., 2021).

In the context of environmental education, two distinct emphases and strategies emerge when teaching about the natural world from a scientific perspective. One approach focuses on the positive impact of nature contact on student well-being and academic performance. The other emphasizes how knowledge of the natural environment fosters more responsible ecological behavior. An often-overlooked yet crucial factor in both approaches is the role that environmental experiences play in shaping a child’s intelligence (in the sense of Piaget and Inhelder) as well as their emotional inclination to preserve what they psychologically consider their own, leading to a sense of stewardship [theories of spatial appropriation by Korosec-Serfaty, 1976 and Pol, 1996, 2002 and attachment theories Lewicka, 2011].

Carrus et al. (2012) demonstrated the positive impact of school activities in naturalized environments on the psychological well-being of young children. However, subsequent research by the same team (Pirchio et al., 2021) yielded less conclusive and even contradictory results, indicating that occasional nature-based activities are insufficient to enhance well-being and promote sustainable lifestyles. Their findings underscore the need for immersive and meaningful nature experiences that go beyond mere cognitive engagement.

The spectrum of EE program typologies is notably diverse in its execution, encompassing variations in both frequency and methodology (García-Vinuesa et al., 2022). It is often integrated as a cross-cutting theme across various subjects, while at other times, specific slots within the curriculum are dedicated to its exploration (Díaz-Pont and Tarragona, 2003, ECEA). Content emphasis may vary depending on whether the focus is on emotional or practical components, or on passive or active engagement. Projects that encourage solution development appear to be particularly effective (Murat, 2015). It is important to recognize that formal education is just one source of environmental input for children and adults. In-depth analysis of the primary and secondary socialization effects of values and models transmitted through increasingly influential virtual networks is crucial, alongside the reality construction undertaken by traditional mass media.

Within this framework, this paper aims to review the findings of three independent studies that delve into the treatment of environmental news in the written press and the pro-environmental involvement of primary, secondary, and university students. The main methodological characteristics of these studies are summarized in Table 1.

**Table 1:** Main methodological characteristics of the studies included in this review.

**Study**	**Reference**	**Population**	**Sample size**	**Period of study**	**Data collection**	**Variables**	**Data Analysis**
Study 1	Castrechini (2008) and Castrechini et al. (2014)	Newspaper news	La Vanguardia: 522. El País: 517. Total: 1039 news.	1992–2006 (pair years only)	Manual collection through microfilm and digital archives.	Number, content and format of the news	Content analysis. Comparative and longitudinal analysis: Chi square, *T*-student and Multiple Correspondence Analysis (MCA)
Study 2	Castrechini et al. (2015) and Pol et al. (2017)	Newspaper news	Year 2004: 53 news. Year 2011: 43 news. Total: 96 news from La Vanguardia.	2004 and 2011	Manual collection through digital archives	Number, content and format of the news	Content analysis. Comparative and longitudinal analysis: Chi square, *T*-student
Study 3	Pol and Castrechini (2013), Castrechini et al. (2015), and Pol et al. (2021)	Primary, high school and university students. Ages: 9–35 years old	Year 2007: 2304. Year 2024: 2487. Year 2023: 2264. Total: 7055 students	Three waves: 1rst study: 2007. 2nd Study: 2014. 3rd Study: 2023.	Questionnaire *ad hoc*, based on the theoretical “Model of four spheres” (Pol, 2000)	Pro-environmental attitudes, believes and behaviours	Analysis of variance (ANOVA)

## 4 From the first and second study: evolution of the image of the environment in media environments

Through a systematic analysis of two prominent Spanish newspapers, namely El País (headquartered in Madrid) and La Vanguardia (based in Barcelona), spanning the period from the Rio Summit to the Johannesburg Summit (1992–2006), a discernible and sustained trend towards augmenting coverage of environmental news emerges. The visual portrayal and the accompanying explications, which encapsulate the SR propagated by the press during these years, undergo a significant transformation, inclusive of the utilization of photographs and graphics, gaining substantial prominence throughout this duration (Castrechini, 2008; Castrechini et al., 2014). When examining absolute figures, the quantity of news coverage essentially doubles from the 1990s to the 2000s. The daily variability inherently hinges on the unfolding of events and occurrences alongside other pertinent variables. Consequently, occurrences like the Prestige catastrophe, as well as significant gatherings such as the Rio Summits, Johannesburg, Kyoto, and the COP meetings, usher in information surges. Yet, irrespective of these specific events, the longitudinal analysis reveals statistically noteworthy disparities affirming a gradual escalation in the daily publication volume of environmental articles each year (χ^2^ = 7273.000; df = 672; *p* < 0.001).

**Figure 1:**
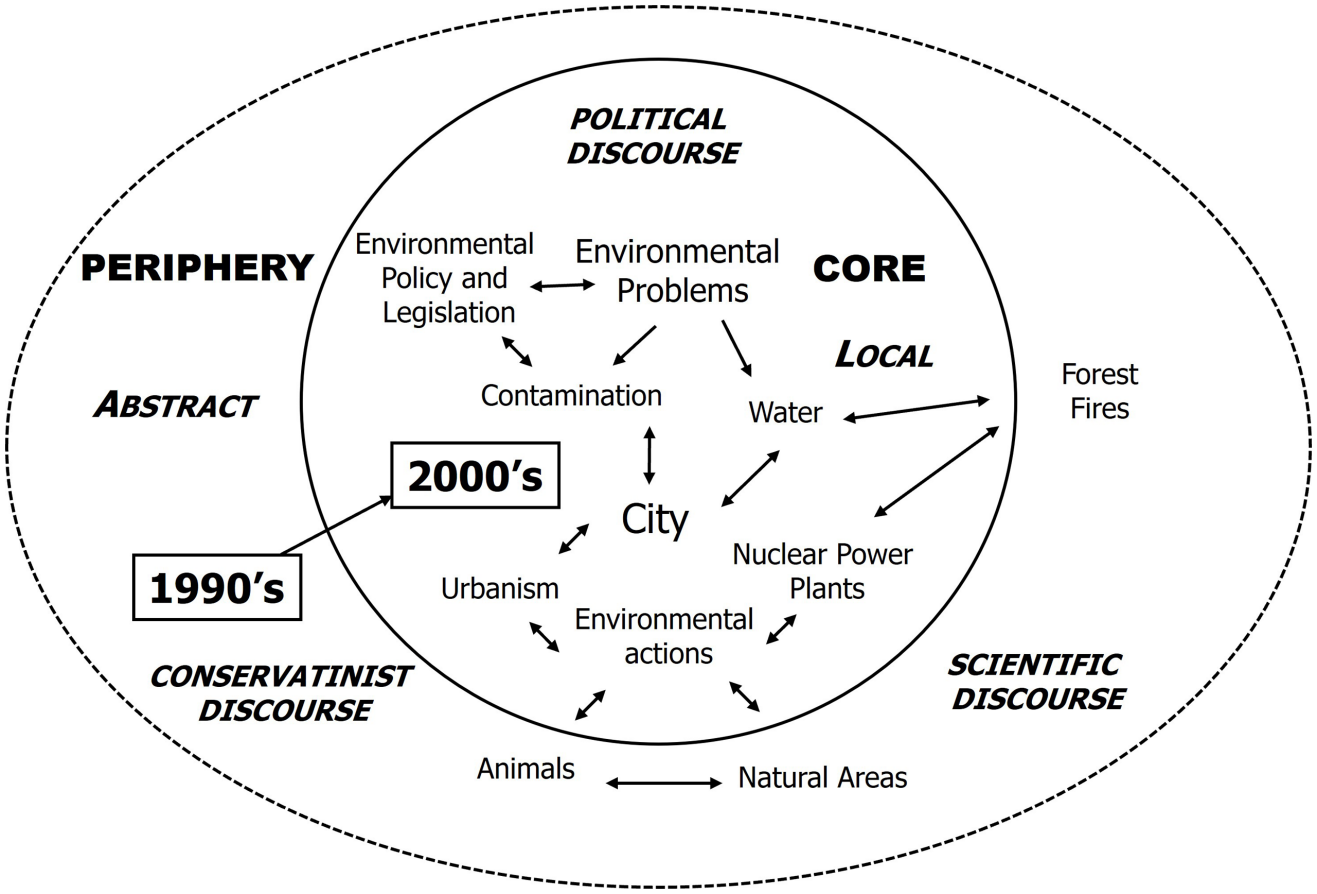
Social representation of the environment in the Barcelona Press (1992–2006) (Castrechini, 2008).

The Figure 1 illustrates the changes in the social representation of the environment between 1992 and 2006 in the Barcelona press. Thematic categories that are closely associated with each other are clustered together on the map, with some occupying the central position and others located on the periphery of social representation. Three distinct types of discourse can be clearly identified: conservationist, scientific, and political. The evolution of environmental discourse is evident in the transition from conservationist to scientific and ultimately to political discourse, highlighting the increasing politicization of environmental issues during this period.

Please note that ‘climate change’ is absent from this figure as it pertains to the findings of Study 1, which focused on the period between 1992 and 2006. During this timeframe, the term ‘climate change’ was still emerging, with discussions primarily centered on the concept of the ‘greenhouse effect.’

Based on the analysis conducted in this study, it is noteworthy that there is instability and variability in the themes, emphasis, slogans, and ways of labeling the environmentally problematic aspects. Until 2006, a significant increase in environmental coverage within mass media was observed; however, this period also witnessed a rise in confusion. A continual shift in emphasis and methods of labelling the highlighted environmental aspects exists, contributing to a sense of distrust among citizens towards environmental and sustainability messages. These shifts in emphasis, often lacking clear explanation or justification, capture the audience’s attention, accomplishing the goal of gaining visibility. However, an unintended consequence emerges: uncertainty propagation and a perception of deceit (Pol, 2011). Leff (1986) asserts that sustainable development cannot materialize without the concurrent development of knowledge; hence, the communication of information transformable into knowledge becomes imperative.

The second research piece we aim to discuss furnishes us with insights into the transformation of the image of AM in media contexts. It delves into a juxtaposition of environmental information preceding, during, and after the 2008 global crisis (Pol et al., 2017). This analysis encompasses 2004 to 2011, encompassing the quantity and nature of news pieces featured in “La Vanguardia.” The study captures the proportional significance of environmental news and delves into a granular exploration of the specific themes covered before, during, and after the crisis.

Globally, a larger number of articles and environmental news pieces were published before the crisis (55.2%) compared to post-crisis (44.8%). However, these differences do not hold statistical significance. Regarding content, the majority (63.7%) pertains to local matters, emphasizing proximity to citizens, while 33.3% of the news covers international, global, or abstract topics. When considering the reference scope of the published information, the contrast between the 2004 and 2011 publications reveals a statistically noteworthy distinction (χ^2^ = 13.128, *p* = 0.001). This divergence was particularly pronounced in 2004, when most environmental publications revolved around local and regional concerns. After the crisis, there was an increase in news items within the International and Economy sections, implying a certain level of abstraction and detachment from the aspects of daily readers’ lives. Moreover, apart from a few generic and abstract reports, brief reports take precedence as the most prevalent journalistic genre during the crisis-post-crisis period. These reports predominantly employ agency news rather than content produced by in-house authors, indicating an allocation of fewer resources and reflecting a diminished prioritization of environmental and sustainability matters.

Furthermore, during the pre-crisis era, the information predominantly encouraged reader involvement and initiative in rectifying environmental concerns. However, in the crisis and post-crisis periods, the disseminated information adopts a notably abstract stance, subtly implying that the intricacies of the environmental issue are so convoluted that it might be wiser for individuals as citizens to refrain from pursuing personal or collective endeavors. Instead, the message suggests adhering to the directives propagated by authoritative entities, presumably substantiated by the “weight of scientific expertise.” (This perspective has been particularly reinforced by the prevailing communication style and official narratives during the pandemic).

These two studies collectively indicate a consistent presence of environmental coverage in the press, although its focal points experience an evolutionary shift over time. Furthermore, during periods of crisis, environmental information tends to diminish in prominence, transitioning from a focus on proximity and citizen engagement to more abstract dimensions that appear distant and less readily identifiable as immediate concerns. Consequently, this phenomenon contributes to what Uzzell (2004) has termed ‘environmental hyperopia’, as discussed in the preceding section. As noted on previous occasions (Pol et al., 2017), this change in emphasis leads to cognitive perplexity and a weakened emotional connection, resulting in reduced personal investment and commitment. This sets the stage for the relevance of the third study we present below.

## 5 From the third study: disruptions in education, awareness and environmental behavior. A longitudinal study

Not too many years ago, the primary challenge of EE was that citizens, particularly children, possessed minimal environmental knowledge required to enhance their ‘awareness’ of the subject. EE programs provided information to address this issue, primarily focusing on the ‘natural’ environment. They facilitated contact with nature whenever feasible, often through rural centers or nature facilities. The aim of imparting knowledge and enabling firsthand experiences was to influence the core components of values, beliefs, attitudes, and behaviors. With many limits, environmental education in the last two decades has provided important achievements and positive examples, particularly when it comes to outdoor education programs that try to go beyond the simple provision of factual information or traditional knowledge transfer in school standard teaching, which also showed the possibility of interesting connections between pro-environmental attitudes and behaviors on the one hand, and subjective well being on the other hand (Carrus et al., 2012; De Dominicis et al., 2017; Pirchio et al., 2021).

More recently, processes of curricular ‘environmentalization’ or ‘sustainability’ have been initiated, entailing the integration and ‘normalization’ of environmental matters as cross-cutting content within formal educational curricula. These efforts align with the Sustainable Development Goals (SDGs). Moreover, periodic scales and questionnaires have been deployed for assessment purposes. A noteworthy example in this context is the New Environmental Paradigm (NEP) proposed by Dunlap and Van Liere (1978), subsequently revised by the same authors (Dunlap et al., 2000; Dunlap, 2008), and applied to diverse populations across various countries (Amérigo and González, 2001; Vozmediano and San Juan, 2005; Ogunbode, 2013; Moyano-Díaz and Palomo-Vélez, 2014). Although widely embraced, this scale has not been exempt from critical scrutiny concerning its application and potential misuse (e.g., Hawcroft and Milfont, 2010).

One notable strength of the NEP scale is its facilitation of cross-national comparisons, offering insights into the progression of environmentalism across different regions of the world—sometimes yielding optimistic outcomes, while at other times, starkly pessimistic ones.

In any case, in the present day, it is a challenging task to encounter young individuals who have not undergone some form of EE in various formats. Knowledge has undeniably expanded; nevertheless, there appears to be a limited substantial enhancement in citizen behavior. Moreover, certain stages in the life cycle seem to be more pivotal than others in this regard. It becomes imperative to question why this is the case and what factors might contribute to this disparity.

An initial study that prompted concern and raised new questions was conducted by Uzzell (1996). Observing children and young individuals before and after their participation in a nature school course or program showed that the participants’ inclination to engage in responsible ecological behavior (CER) had notably decreased. This discovery led Uzzell to validate that, contrary to the intended educational goals of the center’s program, the participants had concluded that the environmental predicament was indeed significant yet incredibly intricate and challenging. Fortunately, they believed there were competent experts, such as their instructors during their time at the center, who could offer solutions. In their view, individual students held no agency to effect change. Additionally, as noted in Uzzell’s subsequent work (2004), he asserts that it is often simpler to express concern for distant environmental issues than to acknowledge those nearer to us—matters that impact our day-to-day existence. This line of thought leads him to introduce the concept of “environmental hypermetropia,” as previously discussed.

Amidst the escalating climate crisis, there has been a notable surge in studies examining the intricate interplay between values, attitudes, and behaviors. These studies span diverse theoretical and epistemological perspectives, often yielding conflicting outcomes. Noteworthy examples among the consulted sources include Corraliza and Martín (1996), Corraliza and Berenguer (2000), Steg and Vlek (2009), Hawcroft and Milfont (2010), Kaiser and Wilson (2019), Kaiser and Wittenberg (2023), and Weis (2023), along with the contributions of Corral-Verdugo (2023). It is important to acknowledge studies that emphasize beliefs and values, such as Bechtel et al. (1999), Dunlap and Van Liere (1978), Dunlap et al. (2000), and Weigel and Weigel (1978), as well as those focus on behaviors (Hess, 1996; Real and García Mira, 2001; Hernández et al., 2002; García Mira et al., 2006).

Against this backdrop and building upon the insights from preceding research endeavors, our research team initiated a study on environmental knowledge and behaviors ^2^ from 2006 to 2007 (Pol and Castrechini, 2013). The outcomes of this investigation yielded unforeseen findings, prompting us to replicate the study a few years subsequently, in 2014 (Castrechini et al., 2015). Furthermore, a third iteration was undertaken, spanning 2022 to 2023 (Pol et al., 2021).

In developing the instrument, a comprehensive review of the literature about scales assessing attitudes, beliefs, and environmentally responsible behaviors, as mentioned earlier, was undertaken. An *ad hoc* questionnaire was meticulously crafted and rigorously validated to suit the various age groups encompassed by the study. The construction of the questionnaire adhered to the 4 Spheres Model (Pol, 2000; Pol et al., 2001). This model delineates four underlying components within attitudes and behaviors: Information and Rationality, Emotionality, Functionality, and social influence processes. A fifth dimension, Directly Expressed Behaviors, was introduced in longitudinal research.

The goal was not to establish a hierarchy of attitudes but rather to encompass the active presence of knowledge regarding ‘what needs to be done and how to do it’—as indicated by the realms of cognition and functionality. This encompasses the willingness to engage in desired behaviors, referred to as the “subjective norm” by Ajzen and Fishbein (1980). Additionally, the emotions influencing the pace or initiation of matters concerning the environment and sustainability—sometimes linked to illusions, desires, and fears—fall within the domain of emotions. Moreover, the impact of external individuals on personal conduct (including imitation, modeling, and the fear of social ridicule for action or inaction) is consolidated under the umbrella of social influence.

A questionnaire was designed to collect data with straightforward inquiries about specific environmental behaviors. This encompassed various actions related to waste management, energy consumption, water usage, and mobility patterns (Pol and Castrechini, 2013).

The approach employed for sample selection, data acquisition, and analysis remained consistent across the three waves, maintaining both the sample size and the participation of training institutions. The only notable divergence pertained to the data collection method: in the initial and second waves, paper-based questionnaires were administered within classrooms, with the cooperation of the teaching staff. Conversely, the third wave adopted an online system utilizing the Qualtrics software while encouraging the participating faculty to engage in classroom responses.

Throughout all three instances, efforts were made to ensure sample uniformity within the four defined age groups: 8–9 years to 12–13 years (primary education); 13–14–15 years (secondary education); 16–17–18 years (baccalaureate and vocational training); and over 18 years (university level). The composition of each study’s samples is outlined in Table 2. Across the waves, the gender distribution remained relatively constant, with an average of 44.7% male participants and 55.3% female participants.

**Table 2:** Composition of the samples of the different applications “Study on the disruption in Education towards Sustainability.”

Age ranges	5–12	13–15	16–17	>18	Total
1rst Study 2006–2007	974	451	320	559	2,304
2nd Study 2014	810	523	464	690	2,487
3th Study 2022–2023	325	761	129	1,049	2,264
Total	2,109	1735	913	2,298	7,055

The questionnaire results underwent thorough statistical analysis. The data from the initial wave unveiled that children aged 8 to 9 through 12 possessed a firm grasp of the prevailing ‘correct’ environmental behaviors at that juncture. These youngsters exhibited greater awareness and espoused more favorable values and beliefs aligned with environmental and sustainability perspectives. Nevertheless, during adolescence, a statistically significant decline in these values was observed (*F* = 35.71, *p* < 0.001, df = 3). This dip was followed by a tendency of recovery as individuals transitioned into the realms of youth and adulthood (university-level sample). Yet, even with this recovery, the attained scores fell short of the commendable benchmarks set by the children’s cohort.

Importantly, it’s worth noting that the adolescents’ scores, albeit diminished, remained remarkably elevated on the scale employed for assessment. Furthermore, the outcomes exhibited nuanced variations contingent upon the specific program and the approach embraced by the educational institution. This divergence stemmed from what was then termed ‘transversality’ or the integration of sustainability education into the core academic curriculum versus ‘exceptionality’ or distinctive actions set apart from the conventional academic routine. These exceptional actions bestowed special prominence upon EE.

The inaugural administration of the questionnaire, referred to as the first wave, yielded conclusions that offer valuable insights into the conventional paradigms of EE—a topic we will revisit in the ensuing discussion. The advent of the global economic crisis in 2008 prompted us to grapple with the task of dissecting its potential impact on predispositions towards sustainability, as well as on the knowledge and behaviors manifested by individuals.

In 2014, the second phase of the same research was conducted, yielding distinct and, once again, unexpected outcomes: the scores of the youngest participants had nearly equaled those of adolescents. However, a perturbation during the adolescent phase persisted (*H* = 138.705, df = 3, *p* < 0.001, Kramer’s *V*: 0.167), engendering fresh inquiries. This downward shift prompted contemplation. What could account for this decline, particularly because educational programs remained unaltered and the sampling centers were consistent with or comparable to the previous wave?

Upon comprehensive analysis, our focus coalesced around the emergence of a novel factor – one absent in 2006–2007: the rapid proliferation of virtual social networks. These platforms, increasingly accessible to minors, exerted a potent influence, not primarily on education, but rather on the intricate socialization processes.

More recently, a new set of conjunctural and structural factors has emerged, providing indications of shifts in the third wave of our study, conducted between 2019 and 2022. This period encompassed the repercussions of the COVID-19 pandemic, exacerbations in the climate crisis, and the escalation of the Russia-Ukraine conflict. The pressing question arises: to what extent have these global factors impacted the general populace’s perceptions, sensitivities, and pro-environmental inclinations?

From 2022 to 2023, the third wave of our research unveils a discernible trend towards decreased overall scores across all age groups. Yet, elucidating the underlying causes has grown considerably more intricate than previous waves, as expounded upon in subsequent sections.

**Figure 2:**
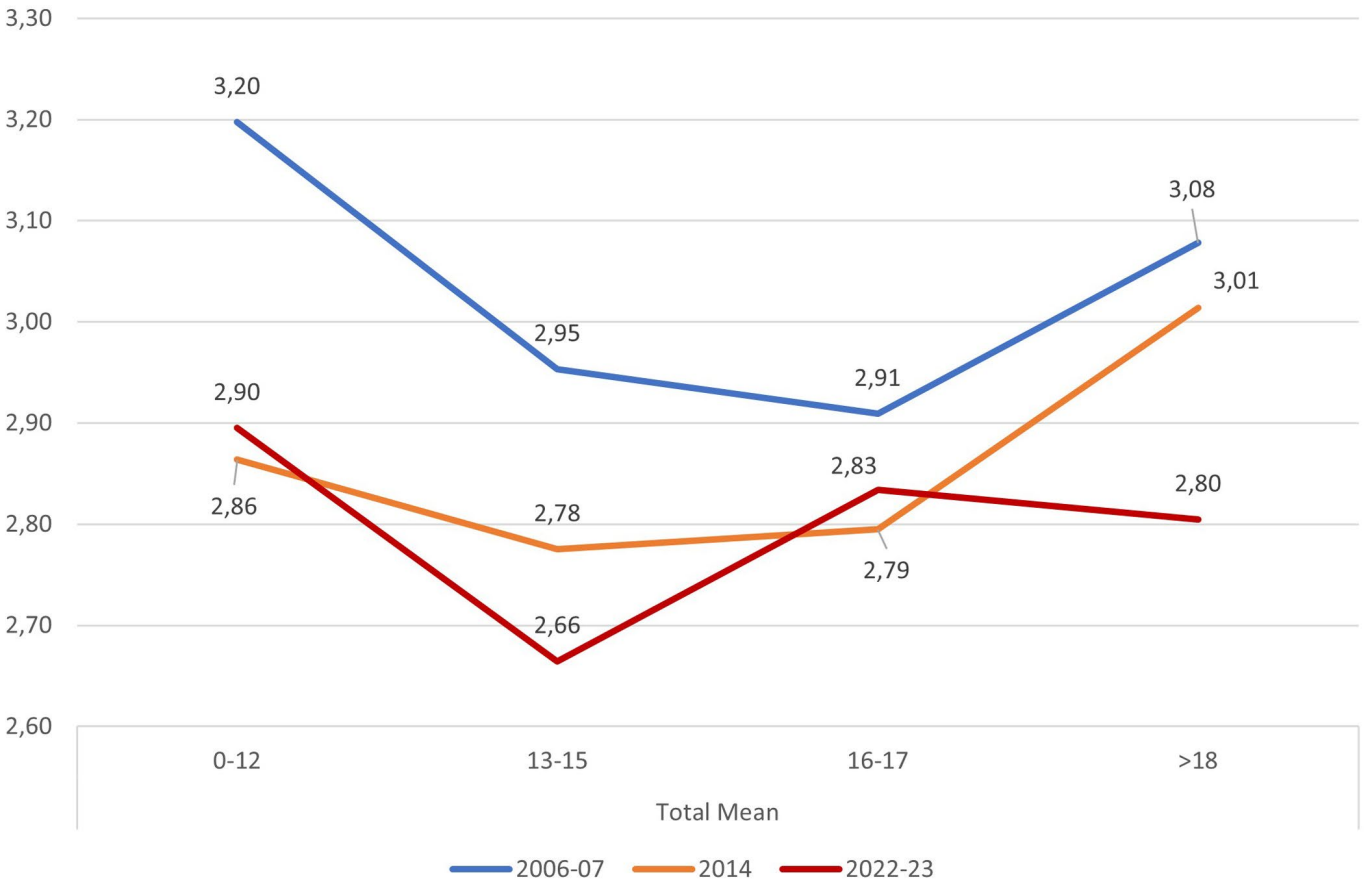
Comparison of scores 2007–2014–2023 by age. The graph represents the results of each of the three applications, showing the scores by age range.

Figure 2 presents the outcomes from the three successive waves of application. The scores for 2006–2007 and 2014 were notably elevated, even at their lowest. However, the 2022–23 wave discloses a discernible decline in scores, which assumes significance when contextualized within the prevailing climate emergency framework and the pervasive information dissemination regarding environmental concerns across media and social networks.

The variations among each wave (application) necessitate distinct explanations for each scenario. The wave from 2006 to 2007 prompted an exploration into the reasons behind the phenomenon termed ‘disruption’ in EE, as noted by Pol and Castrechini (2013). The wave from 2014 brought to the forefront inquiries regarding alterations within the social context, shedding light on why the performance of the youngest group decreased even more significantly than that of the adolescents. The data from 2022 to 2023 raises a pertinent question: why, during a period characterized by heightened concerns about sustainability and the climate emergency, do global scores experience a notable decline?

Furthermore, the outcomes allow us to reaffirm a fact widely recognized in environmental psychology and other social and behavioral sciences yet frequently overlooked, resulting in misguided educational and training strategies. It’s important to note that information and/or knowledge alone does not guarantee appropriate, consistent behavior, nor do they effectively alter habits or routines. In essence, while information is essential, it remains insufficient. Moreover, this phenomenon can be observed (particularly within the university sample), where specific training can paradoxically lead to a rebound effect in behavior or, at the very least, a degree of desensitization or relativization of the problem. This observation holds particularly true when the recipients are prospective professionals in environmental technologies.

In the initial wave, the youngest participants achieved the highest scores and exhibited the greatest responsiveness to the social norm. This tendency can likely be attributed to their evolutionary or maturation stage, a concept expounded by Piaget and Inhelder (1969). According to these scholars, children aged 9 to 11 find themselves in the concrete operational stage, progressing to the abstract operational stage from ages 11 to 12. Nevertheless, this perspective – when applied to EE as per the viewpoints of Spencer and Darvizeh (1981) – resulted in a significant underestimation of the competencies and environmental aptitude inherent in younger children. Concurrently, it led to a certain disregard for the more contentious aspects of adolescence and its associated crises.

During adolescence, an observable inclination toward deliberately transgressive behaviors becomes apparent (Funes, 2010) – a tendency, it’s important to note, that’s prevalent among all individuals. This penchant for transgression during adolescence stems more from the evolutionary crisis inherent to this phase, closely tied to the imperative of self-identification and differentiation rather than from inadequacies within training programs. This phenomenon is closely interwoven with external factors beyond the school environment. Moreover, it is pertinent to contemplate the extent to which the incorporation of environmental values advocated by EE initiatives and certain media influences the perception of these values as “formal and official tenets of the societal framework.” Adolescents often oppose these values during identity crises as they search for meaning. Therefore, the disruption experienced during adolescence is more aligned with the inclination to ‘transgress’ rather than a dearth of training, information, or ignorance concerning desirable behavioral norms.

Within the European context, Grønhoj and Thøgersen (2009) similarly observed a phenomenon wherein adolescents display lower environmental commitment compared to their parents – an occurrence the authors call the “generation gap.” While attitudes towards the environment generally skew positive, a discernible discrepancy exists in the manifestation of values and behaviors. This discrepancy is ascribed to the challenges inherent in the developmental stage experienced by adolescents, during which the prevailing priority values diverge from those upheld by adults.

In the initial two studies, university students exhibit more favorable behavior than adolescents, although they cannot “attain” the levels observed in primary school children during the 2006–2007 period. However, in the third wave, their scores remain stagnant at the same level as the adolescent cohort. Additionally, several unexpected trends become apparent in the disaggregated analyses. For instance, attitudes and behaviors concerning waste management fare worse than the broader sample. Furthermore, a noteworthy observation emerges from 2007: the university (or institution of study) that allocated greater time and resources to environmental management and the restructuring of their curricula to provide targeted sustainability education to students yielded inferior outcomes in values tied to civility and processes of social influence, as opposed to other universities.

The resurgence of scores within the university setting appears to hinge more on contextual factors that enhance these values and behaviors rather than solely on the training imparted. Furthermore, this resurgence seems to be counteracted or reduced by a sense of familiarity, knowledge, or a perception of control over environmental challenges and technologies – factors poised to shape their forthcoming professional endeavors.

### 5.1 Transversality vs. exceptionality?

The findings raise suspicions that the ‘normalization’ of environmental training as a pervasive value in certain school and academic curricula causes these values to go unnoticed. Conversely, the exceptional nature of specific actions appears to enhance their memorability and serve as stronger catalysts for the expression of current behavior. The analyses indicate notable disparities between students who recall participating in environmental training activities and those who do not.

Moreover, based on the data provided to educational institutions, the count of respondents who have completed environmental and sustainability training significantly surpasses the count of those who remember having engaged in such activities. This trend aligns with observations from other studies in different contexts (e.g., labor) and various countries, wherein ‘mainstreaming’ (typically perceived as the earnest and desirable approach) proves less effective than ‘exceptionalism’ – referring to specific acts with a certain festive or folkloric quality, often criticized as superficial and shallow.

A similar scenario unfolds concerning awareness of environmental issues or policies within organizations that have attained an EMAS certification (a European system somewhat akin to ISO 14,000). This occurs when they employ an “integrated management system,” resulting in a dilution of environmental focus. Conversely, this dilution is absent when a specialized environmental management system is employed (Pol et al., 2006). Comparable outcomes emerged in other contexts, such as Brazil (Bolzan de Campos, 2008; Bolzan de Campos and Pol, 2009) and Mexico (Manzano, 2016; Peña, 2016).

The suspicion that specific training might lead to a rebound effect on behavioral aspects while failing to instill environmentally suitable habits and behaviors challenges the current sustainability model implemented in schools and colleges. These suspicions are grounded in empirical observations; however, they warrant further dedicated studies to provide more conclusive insights. Nonetheless, a mounting number of indications are pointing in this direction. This is a significant motivator behind our undertaking of this longitudinal study.

## 6 Discussion

As observed in the results of the various synthesized investigations, distinct interaction and interdependence exist among the conceptual axes explored in each study. This point becomes even more evident when we consider the outcomes of longitudinal research. The fluctuations in the results across the three waves of knowledge and predisposition analysis toward more ecologically respectful behavior cannot be comprehended without accounting for the shifts in communication technologies, alterations in perceptions or societal portrayals of the environment, sustainability, and the climate crisis.

Moreover, understanding these changes requires acknowledging the evolving nature of socialization processes and the influential sources or stimuli that significantly shape this progression. Notably, this aspect has strikingly vanished from scientific literature in recent decades, encompassing various fields such as psychology (including general, social, and environmental psychology), education, and sociology.

As demonstrated by Castrechini (2008), the media’s coverage of environmental issues experienced a substantial increase between 1992 and 2006. Its content transitioned from primarily scientific to political and legislative during the 1990s. This transformation can be largely attributed to the influence of the Brundtland report (Brundtland, 1987) and the Porto Alegre Earth Summit (1992), which emphasized the need to establish appropriate legislative bodies and encouraged active engagement from environmental movements. This involvement expanded to encompass not only grassroots organizations but also governmental agencies.

Moving into the 2000s, the discourse surrounding environmental issues further evolved, taking on a more abstract tone. There was a heightened focus on conservation and scientific dialogues while still retaining a strong emphasis on the importance of citizen engagement.

In the second analyzed investigation, the data again reveals a significant shift when comparing the periods before and immediately after the economic crisis of 2008. The pre-crisis phase focused on empowerment, consistently emphasizing the need for people’s involvement and commitment to initiate actions. However, the post-crisis period witnessed a change in the positioning and underlying message of the information, even though the volume of information remained consistent with the previous phase. Following the crisis, environmental information began to be situated within more abstract contexts, conveying the message that the issue is of such gravity and complexity that personal, social, or community initiatives should not be pursued. Instead, the emphasis shifted to macro-level politics, economics, and “science,” the narrative urged compliance with these broader frameworks. This shift led to a sense of helplessness, reliance, and impotence. To a large extent, it generated a sense of deception, especially because the scientific discourse on environmental issues evolves over time, with emphases and explanations that are sometimes contradictory, thus running the risk of losing credibility, as mentioned above.

This effect aligns with the findings of Uzzell (1996, 2004), who observed a discouraging impact on children after participating in a nature school. These children perceived the environmental issue as so intricate that grasping it was challenging, and they considered themselves fortunate that there were experts who understood it comprehensively, thus alleviating the need for a personal concern. In the context of the nature school, this outcome starkly contradicted the institution’s intended purpose. However, concerning media coverage, whether this outcome is unintentional becomes less apparent. Both mass media and social networks play pivotal roles in constructing narratives and shaping SR recall Ruiz’s (2015) characterization of “communicators” as symbolic “architects” of realities. This is particularly pertinent within the broader context of profound societal shifts catalyzed by the 2008 crisis on both local and global scales. Consequently, if collective actions by individuals can contribute to addressing environmental challenges, they also possess the potential to confront and transform various socioeconomic and political dimensions of local and global societies. Yet, these latter implications might not align with certain segments’ interests.

If we delve further into the past, the evolution of EE, often considered the cornerstone for catalyzing shifts in individuals’ attitudes and behaviors, is worth examining. As we have mentioned during the 1970s, it emerged as a ‘revolutionary’ endeavor championed by nascent environmental movements. However, as we progressed into the late 1980s, the 1990s, and beyond, it transitioned to being endorsed by institutional bodies. This change in the source of promotion brings forth pertinent discussions surrounding the credibility and trustworthiness of the message disseminators (Newman et al., 2021). The shift to ‘institutional’ sources, associated with power and authority, to a significant extent, explains the notable decline in adolescent engagement observed in the study conducted between 2006 and 2007. Moreover, this trend of diminishing involvement is consistent with downward trajectories witnessed in subsequent studies.

The noteworthy decline in the scores achieved by the youngest participants in 2014, a decline surpassing the scores of adolescents in 2006–2007, persists into the 2022–23 timeframe. Moreover, the third wave saw a universal drop in overall scores across all age groups. While we do not primarily attribute this phenomenon to deficiencies in formal classroom education, it appears to be influenced by the emergent role of virtual social networks and media as powerful agents of socialization. This role began to take shape in 2006–2007 and has since increasingly assumed the role of a primary socialization agent, often overshadowing other traditional agents, particularly in the case of children and adolescents (Stasova and Khynova, 2012).

As we alluded to earlier, literature regarding the impact of social networks on socialization remains relatively scarce. The existing literature concentrates more on the potential “addictive” and pathological effects on adolescent development (Malo Cerrato et al., 2023) rather than conducting comprehensive analyses of the content, values, or behavioral patterns propagated by these networks. Nevertheless, it is these patterns that wield considerable influence over subtle processes of socialization. This situation underscores a significant pending challenge: a thorough and rigorous examination of the transformed landscape in transmitting values, personal paradigms, and behavioral norms. This exploration needs to be undertaken in a future context and within the immediacy of the present. Furthermore, this imperative extends beyond environmental values and behaviors, encompassing a contribution to comprehending the escalation of aggressive behaviors and gender-based violence among children – a distressing trend that appears to be proliferating across society.

Another dimension illuminated by our data pertains to educational strategies and the standardization of environmental knowledge within formal educational curricula (García-Vinuesa et al., 2022). As early as the 2006–2007 sample, a striking trend emerged. Students attending institutions with fully integrated EE content and activities seemed to recall these experiences less or exhibited diminished awareness of their participation compared to their counterparts in schools where pro-environmental initiatives were positioned as distinctive or exceptional occurrences. This pattern persisted through the subsequent two waves, despite concerted endeavors to enhance EE programs (Martí, 2003; Rodrigo-Cano et al., 2019).

A comparable contradiction emerges in the context of the university samples across the three waves, necessitating an alternate theoretical interpretation. Our analysis compares what we have classified as education directly engaged with environmental concerns versus non-environmentalized education. Strikingly, paradoxically, students categorized as “non-greened” consistently achieve higher scores across the three waves than their peers enrolled in environmentally integrated or actively engaged environmental coursework.

As demonstrated in the results section, the university-aged cohort in the first two waves reveals a resurgence concerning environmental awareness compared to their adolescent counterparts. However, this improvement does not hold in the 2022–2023 sample. Yet, across all three instances, a comparable phenomenon akin to that observed in primary and secondary education manifests, albeit with distinctive nuances. Individuals pursuing non-environmentalized academic tracks consistently score more than those directly engaged with environmental subjects or issues. In the initial and subsequent waves, university students generally display a slightly heightened sensitivity to environmental concerns, likely influenced by the impact of narratives, imagery, and SR that accentuate the topic. This heightened sensitivity will diminish by 2023.

However, a striking trend common to all three waves is the lower scores attained by those actively engaged with environmental subjects. Regarding a theoretical interpretation, this pattern might resemble the concept of objective risk versus risk perception. Exposure or acclimatization to risk can reduce perception, even when the objective risk remains substantial (Lima et al., 2005). Additionally, a degree of cognitive dissonance (in Festinger’s sense) might emerge between being trained to rectify or transform environmental issues and perceiving the situation as exceedingly critical. A prevailing sense of self-assuredness in their capacity to positively influence environmental improvement often supersedes the belief that, as experts, they might be incapable of rectifying the situation. This inclination aligns with the trend described by Fointiat (1998) and Fointiat et al. (2011) in their exploration of ‘rationalization’ instead of ‘rationality.’

All of these insights collectively lead us to conclude that a paramount and integral aspect lies in enhancing information processing and communication management. These factors are foundational to the psychological and societal construction of sustainability. The advancement of knowledge concerning sustainability and the climate crisis is inherently dynamic and subject to ongoing progress. This becomes clear with the phenomenon of solastalgia and which could be understood as the feeling of sorrow that comes with the loss of previous environmental conditions (Albrecht et al., 2007). The mismanagement of this information engenders scenarios rife with message inconsistencies, ultimately breeding uncertainties, fostering distrust, cultivating a sense of deception, and contributing to a surge in eco-fatigue (Pol, 2000) and eco-anxiety (Albrecht, 2011).

Thus, the perspective pioneered by Festinger, which still garners adherents, elucidated how individuals tend to incorporate only information that aligns with their pre-existing beliefs (rationalization). This phenomenon hinders implementing initiatives to foster sustainable behaviors, as it complicates efforts to prompt actions that foster sustainability-aligned conduct.

This rationalization process similarly influences the inherent construction of realities, narratives, and social representations stemming from human behavior, potentially exacerbating confusion. Moreover, scrutiny of socialization processes is imperative, spanning both the formative years of childhood and the adult population. In the ICTs age, images and value frameworks proliferate, obscuring the origins of these constructs, making it challenging to ascertain definitively who authored them, the methods of creation, and the underlying motivations. At best, we can discern the sender of these materials.

## 7 Conclusion and new challenges

Our review of literature from various fields, combined with our own synthesized research findings, has enabled us to propose a series of relationships and explanatory statements that shed light on aspects of contemporary reality. However, we must mention that the main limitation of this review has focused on some specific studies from the authors’ own research output. A broader and more diverse review could have provided a more contrasted view. Additionally, due to the inherent difficulty in establishing clear causal relationships that do not oversimplify or distort reality, many of our assertions and conclusions require further theoretical and empirical analysis.

A crucial area of research lies in examining, contrasting, and empirically quantifying the values, personal models, and behavioral patterns being disseminated through social networks, particularly among young people and children. Understanding these influences is essential not only for shaping environmental values and behaviors but also for deciphering the rise of aggressive behavior and gender-based violence among youth.

Another element demanding ongoing attention is the evolving image of the environment, sustainability, climate change, and related issues constructed by mass and social media. This knowledge is essential for developing effective change strategies and education programs. Moreover, understanding and evaluating the key effects of socialization, often overlooked in current theoretical and empirical analyses, is particularly important in this context.

We have explored how current dynamics can enhance empowerment or create “induced” situations of learned helplessness (in Seligman’s sense), facilitated by relational alterations (losses) induced by ICTs and especially social media. We have detected and described a fundamental implicit change in the contents and strategies of environmental communication before and after the 2008 global economic crisis.

However, we must continue to analyze the prevalence of specific communication strategies in the present and future. Assessing the overall credibility of environmental information reaching the public requires examining the self-interested use and abuse of such information, for instance, in greenwashing and in explicit and subtle economic and political maneuvering. This underscores the importance of environmental information management. Poor information management leads to contradictions that generate uncertainty, a sense of deception, loss of credibility, and increased eco-fatigue, eco-anxiety and solastalgia.

Further research is needed to investigate why individuals actively working on environmental issues or pursuing related education tend to score lower in some surveys of environmental concern. To what extent is this a personal strategy to reduce cognitive dissonance (in Festinger’s sense) between working or training to address environmental problems and perceiving the situation as very serious or irreversible? Does self-confidence in one’s ability to positively influence environmental correction outweigh the belief that, as technicians, one cannot fully remedy the situation?

In conclusion, while studying the relationships between values, attitudes, and behaviors from a psycho-socio-environmental perspective is essential, we must also consider the interconnected processes of reality construction, mass media’s influence on shaping meaning, networks’ impact on societal socialization, and the challenges posed to traditional education by technological, structural, socio-economic, and political shifts. These factors ultimately influence what is taught, what is not, what is recognized and prioritized as “science,” and how the emphasis placed on specific aspects or processes, intentionally or unintentionally, affects the credibility of sources, public trust, and the willingness to engage in ecologically responsible and active behaviors. Understanding these dynamics is crucial for preventing a sense of powerlessness stemming from “induced” helplessness, socio-economic factors, and the questionable effects of emerging technologies.

## Author contributions

EP: Conceptualization, Funding acquisition, Methodology, Project administration, Writing – original draft, Writing – review & editing. AC-T: Methodology, Project administration, Writing – original draft, Writing – review & editing. IP-C: Investigation, Methodology, Writing – original draft, Writing – review & editing. CC-M: Data curation, Formal analysis, Visualization, Writing – review & editing.

## Footnotes

^1^COP is the acronym for the “Conference of the Parties,” Annual Summit carried out by the United Nations Framework Convention on Climate Change (UNFCCC) where they bring together the 196 countries that make up the Parties.

^2^When the study began, the research team was linked to the Catalan Network for Research in Education for Sustainability (www.Edusost.cat), currently the research group is part of the consolidated Research Group of the University of Barcelona in Psychology Social, Environmental and Organizational (PSICOSAO) (http://www.ub.edu/grc_psicosao2/index.php?lang=es).
